# Rapid Insulin-Dependent Endocytosis of the Insulin Receptor by Caveolae in Primary Adipocytes

**DOI:** 10.1371/journal.pone.0005985

**Published:** 2009-06-19

**Authors:** Siri Fagerholm, Unn Örtegren, Margareta Karlsson, Iida Ruishalme, Peter Strålfors

**Affiliations:** Division of Cell Biology, Department of Clinical and Experimental Medicine and Diabetes Research Centre, University of Linköping, Linköping, Sweden; Karolinska Institutet, Sweden

## Abstract

**Background:**

The insulin receptor is localized in caveolae and is dependent on caveolae or cholesterol for signaling in adipocytes. When stimulated with insulin, the receptor is internalized.

**Methodology/Principal Findings:**

We examined primary rat adipocytes by subcellular fractionation to examine if the insulin receptor was internalized in a caveolae-mediated process. Insulin induced a rapid, t_1/2_<3 min, endocytosis of the insulin receptor in parallel with receptor tyrosine autophosphorylation. Concomitantly, caveolin-1 was phosphorylated at tyrosine(14) and endocytosed. Vanadate increased the phosphorylation of caveolin-1 without affecting insulin receptor phosphorylation or endocytosis. Immunocapture of endosomal vesicles with antibodies against the insulin receptor co-captured caveolin-1 and immunocapture with antibodies against tyrosine(14)-phosphorylated caveolin-1 co-captured the insulin receptor, demonstrating that the insulin receptor was endocytosed together with tyrosine(14)-phosphorylated caveolin-1. By immunogold electron microscopy the insulin receptor and caveolin-1 were colocalized in endosome vesicles that resembled caveosomes. Clathrin was not endocytosed with the insulin receptor and the inhibitor of clathrin-coated pit-mediated endocytosis, chlorpromazine, did not inhibit internalization of the insulin receptor, while transferrin receptor internalization was inhibited.

**Conclusion:**

It is concluded that in response to insulin stimulation the autophosphorylated insulin receptor in primary adipocytes is rapidly endocytosed in a caveolae-mediated process, involving tyrosine phosphorylation of caveolin-1.

## Introduction

The insulin receptor, similarly to many other hormone receptors, is internalized by endocytosis upon ligand binding [1]. Endocytosis of hormone receptors has been associated with proteolytic degradation of the ligand or receptor and ligand, causing downregulation of the receptor leading to reduced responsiveness of cells and tissues to the hormone [2]–[5]. The opposite has also been reported, that receptor endocytosis serves to ensure sustained signaling. Endocytosis of hormone receptors has also been suggested to be part of the signaling process, thus providing access to intracellular proteins and structures for the active receptor, which can contribute to the pleiotropy in a hormonal response, reviewed in ref [6]. Evidence in support of this for the insulin receptor has accrued [7]–[14], but there are also reports to the contrary [15], [16] and conclusive evidence for a direct role of endocytosis of the insulin receptor in insulin signaling is lacking.

The insulin regulated internalization of insulin receptors has been shown to depend on insulin receptor autophosphorylation [8], [17], [18], but to be independent of the downstream phosphorylation or activation of insulin receptor substrate (IRS) or phosphatidylinositol–3 kinase in CHO cells [18]. Most work has concentrated on endocytosis of the insulin receptor through the clathrin-coated pit-mediated pathway [1], [19], but reports have also suggested other pathways for insulin receptor internalization [20], [21].

In adipocytes the insulin receptor has, by immunogold electron microscopy, biochemical isolation, and functional analyses, been demonstrated to be localized to caveolae in the plasma membrane [22]–[27]. There are, however, reports that fail to find the receptor in caveolae [28]–[30], which may result from examination of other cell types and different methodologies. The insulin receptor is, for example, soluble in detergent [22], whereas many caveolae localized proteins, including caveolin, are insoluble under the same conditions. Caveolae are invaginations of the plasma membrane that are involved in organizing signaling across the membrane. Although still a matter in debate, caveolae have also been implicated in endocytosis [31], [32]. It has been demonstrated that the majority of caveolae are static at the plasma membrane with a low rate of constitutive endocytosis. However, endocytosis can be induced, for review see ref [33]. The primary structural protein of caveolae, caveolin, exists in the three major isoforms caveolin-1, -2, and -3. Caveolin-1 and -2 are more or less ubiquitously expressed, while caveolin-3 is muscle specific. The role of caveolin in endocytosis is not clear. Caveolin has been demonstrated to stabilize caveolae at the plasma membrane, while in the absence of caveolin caveolae form but are rapidly endocytosed [34]. Together with caveolin-1 interaction with actin filaments [35], this may explain the relative inertness of caveolae at the plasma membrane. It also implies that caveolin-1 may be critical for regulation of endocytosis. Indeed, caveolins are multiply phosphorylated proteins and specifically phosphorylation of caveolin-1 at tyrosine(14) by src-kinase has been shown to be involved in endocytosis [36]–[38].

We wanted to examine the involvement of caveolae in the early phase of insulin-stimulated endocytosis of the insulin receptor in primary adipocytes. Primary rat adipocytes have a very thin (200–500 nm) cytoplasmic space between the lipid droplet and the plasma membrane, which makes it difficult to use immunofluorescence microscopy to distinguish cytosolic processes from events at the plasma membrane. Herein we have used subcellular fractionation to demonstrate that in primary adipocytes insulin stimulation induced rapid insulin receptor endocytosis by way of caveolae.

## Results

### Endocytosis of the autophosphorylated insulin receptor

**Figure 1:**
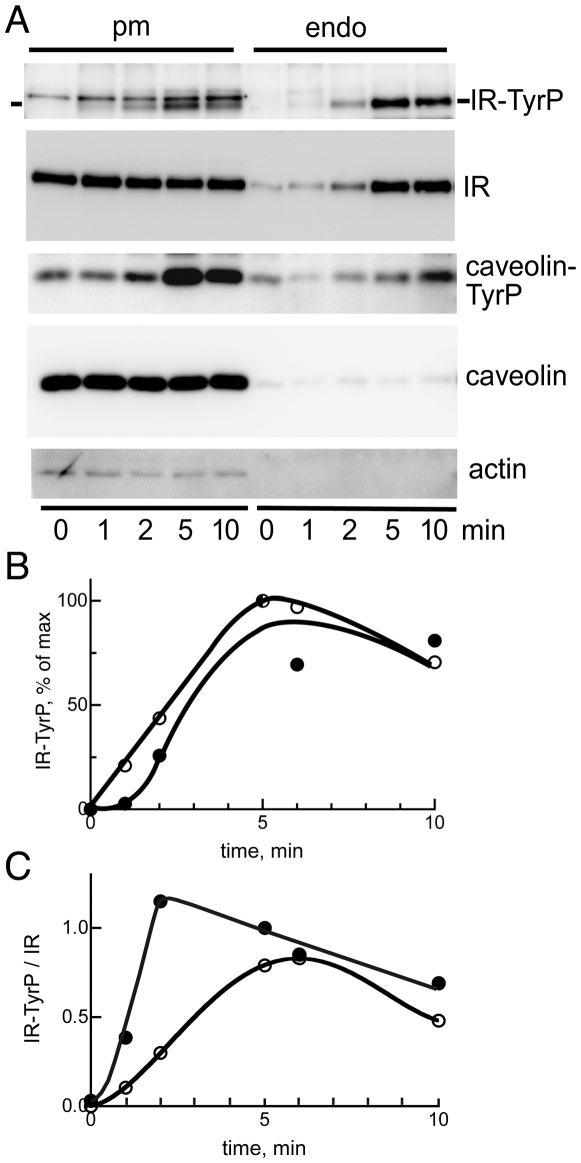
Phosphorylation and endocytosis of the insulin receptor. Isolated adipocytes were incubated with 100 nM insulin for the indicated time, when cells were homogenized and plasma membrane (pm) and endosomal (endo) fractions isolated. A. Equal amounts of protein (5 µg) were subjected to SDS-PAGE and immunoblotting with (from the top) antibodies against phosphotyrosine (indicated is the insulin receptor, IR-TyrP, identified by its comigration with insulin receptor protein on the same blot), insulin receptor β-subunit (IR), tyrosine(14)-phosphorylated caveolin-1 (caveolin-TyrP), caveolin-1, and actin. B, C. Time-course for internalization of phosphorylated insulin receptor. Two experiments were quantified and the average of the two is presented as percent of maximal insulin effect on phosphorylation of the insulin receptor at tyrosine (IR-TyrP) (B) or as a ratio of IR-TyrP to the amount of insulin receptor β-subunit protein (IR) (C), in the plasma membrane (open symbols) and endosomal fraction (closed symbols).

Following addition of insulin to isolated rat adipocytes the insulin receptor in the plasma membrane fraction was rapidly autophosphorylated and reached maximal phosphorylation within 5 min (Fig. 1A). In close parallel the receptor was internalized and recovered in an intracellular/endosome fraction, reaching maximum internalization within 5 min (Fig. 1A). Only a fraction of the receptors were internalized; 91±7% (mean±SE, n = 11 separate experiments) of insulin receptors remained in the plasma membrane after insulin stimulation for 10 min. The total amount of receptors in the cells did not change during 10 min of stimulation with insulin (not shown). Little insulin receptors were recovered in the endosome fraction in the absence of insulin stimulation (Fig. 1). This demonstrates that insulin receptors recovered in the endosome fraction after insulin stimulation were internalized, and therefore cannot come from contaminating plasma membrane fragments or caveolae that have been torn off the plasma membrane during the homogenization and subcellular fractionation of the adipocytes; and that we therefore can refer to this fraction as an endosome fraction. Hence, insulin receptors in the endosome fraction correspond largely to endocytosed receptors.

After a short lag endocytosed receptors became phosphorylated in parallel with receptors in the plasma membrane (Fig. 1B), indicating rapid internalization of autophosphorylated receptors. Comparing the ratio of phosphorylated to total amount of receptors in the plasma membrane and in the endosome fraction, revealed that endocytosed receptors were immediately phosphorylated to a higher extent than in the plasma membrane (Fig. 1C). This indicates that phosphorylated receptors were preferentially or exclusively endocytosed, similarly to what has earlier been reported [10]. The amount of receptors remained at an elevated level in the endosome fraction, while the phosphorylation of the receptors peaked at about 2 min after which it declined, in the continued presence of insulin (Fig. 1). This indicates that the receptors were rapidly dephosphorylated by an intracellular phosphotyrosine protein phosphatase.

Caveolin-1 was phosphorylated on tyrosine(14) in response to insulin at the plasma membrane (128±10% of control, n = 14 separate experiments; p<0.05 by Student's t-test) (Fig. 1), essentially as has been described for 3T3-L1 adipocytes [29]. Tyrosine(14)-phosphorylated caveolin was also internalized to the endosomal fraction (135±15% of control, n = 12 separate experiments, p<0.05 by Student’s t-test) in conjunction with the insulin receptor (Fig. 1). As only a fraction of the insulin receptors was endocytosed, an even smaller fraction of total plasma membrane caveolin-1 was endocytosed, cf. ref [39], explaining the small and variable effect on the caveolin-1 fraction recovered in the endosomal fraction.

**Figure 2:**
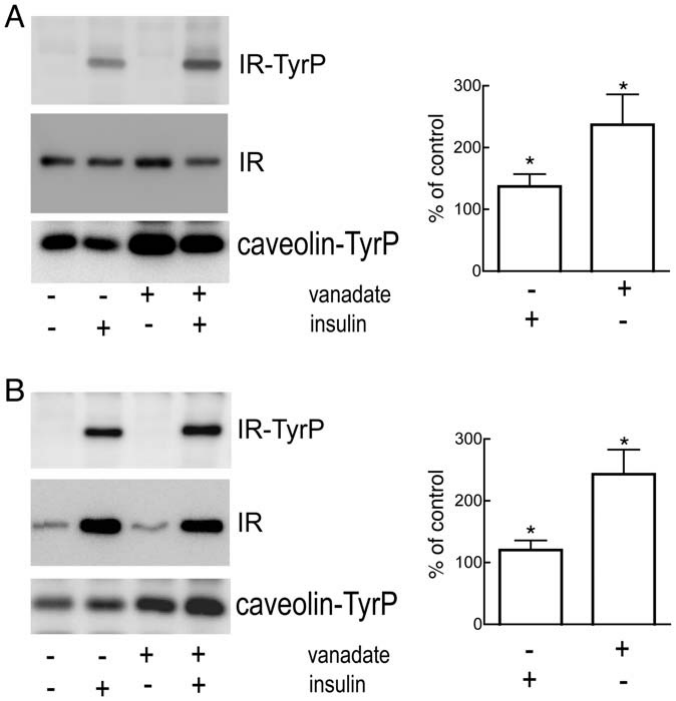
Effects of vanadate on endocytosis of the insulin receptor. Isolated adipocytes were incubated with or without 2 mM Na-ortho-vanadate for 20 min, when insulin at 100 nM was added for another 10 min, as indicated. Cells were then homogenized and plasma membrane (A) and endosomal (B) fractions isolated. Equal amounts of protein were subjected to SDS-PAGE and immunoblotting with (from the top) antibodies against phosphotyrosine (indicated is the insulin receptor, IR-TyrP), insulin receptor β-subunit (IR), and tyrosine(14)-phosphorylated caveolin-1 (caveolin-TyrP). Bar graphs show the quantitation of tyrosine(14)-phosphorylated caveolin-1 as percent of controls without additions, in 7 separate experiments, mean±SE. *, p<0.05 by Student’s t-test.

The general phosphotyrosine protein phosphatase inhibitor sodium ortho-vanadate enhanced caveolin-1 phosphorylation at tyrosine(14) at the plasma membrane to a much larger extent than insulin (Fig. 2A). Ortho-vanadate did, however, under these conditions, not affect the state of phosphorylation of the insulin receptor (Fig. 2A). Likewise, ortho-vanadate did not affect insulin receptor endocytosis, although tyrosine(14)-caveolin was internalized to the endosomal fraction (Fig. 2B). Neither insulin nor vanadate caused a significant reduction of the amount of caveolin protein in the plasma membrane, indicating that only a minor fraction of plasma membrane caveolin was phosphorylated and endocytosed in response to either agent. The inhibitors of protein tyrosine kinase phosphorylation genistein (100 µM, 20 min preincubation) or herbimycin (4 µg/ml, 20 min preincubation) had only little or no effect on insulin stimulation of insulin receptor autophosphorylation, cf. ref [40], or endocytosis (not shown).

### Endocytosis of the insulin receptor via caveolae

**Figure 3:**
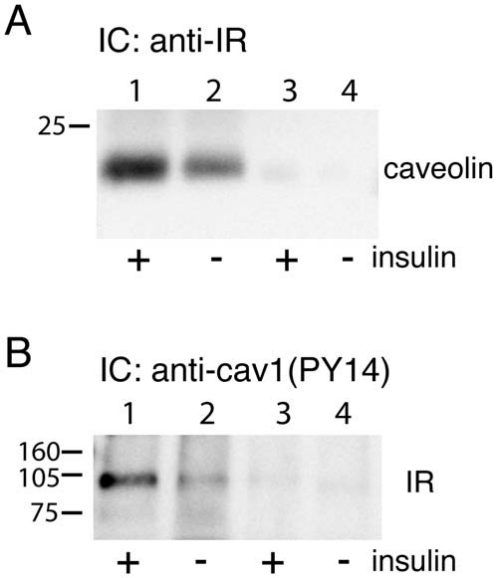
Immunocapture of vesicles in endosome fraction. Isolated adipocytes were incubated with or without 100 nM insulin, as indicated, for 10 min, when cells were homogenized and the endosomal fraction isolated. A. The endosomal fraction was incubated with antibodies against insulin receptor β-subunit (anti-IR) and immunocaptured (IC) (lanes 1 and 2). B. The endosomal fraction was incubated with antibodies against tyrosine(14)-phosphorylated caveolin-1 (anti-cav1(PY14)) and immunocaptured (lanes 1 and 2). Equal amount of protein was subjected to SDS-PAGE and immunoblotting with antibodies against caveolin-1 (A) and insulin receptor β-subunit (IR) (B). In the absence of primary antibody no antigen was immunodetected (lanes 3 and 4). Positions of reference proteins are indicated (kDa).

To examine whether the insulin receptor was internalized in the same membrane vesicles as caveolin-1, we used antibodies attached to magnetic beads to immunocapture insulin receptor-containing membrane vesicles from the endosome fraction. As determined by SDS-PAGE and immunoblotting the insulin receptor-captured vesicles contained caveolin-1, 10% (average of two experiments) of caveolin-1 in the endosome fraction was co-captured (Fig. 3A). In the reverse experiment we immunocaptured tyrosine(14)-phosphorylated caveolin-1-containing vesicles from the endosome fraction. SDS-PAGE and immunoblotting revealed that the immunocaptured vesicles contained the insulin receptor, 53% (average of two experiments) of insulin receptor in the endosome fraction was co-captured (Fig. 3B). In the absence of primary antibodies during the immunocapture procedure, no insulin receptor or caveolin-1, respectively, was captured (Fig. 3). Thus, in the endosome fraction the insulin receptor was recovered in the same membrane vesicles as caveolin-1.

**Figure 4:**
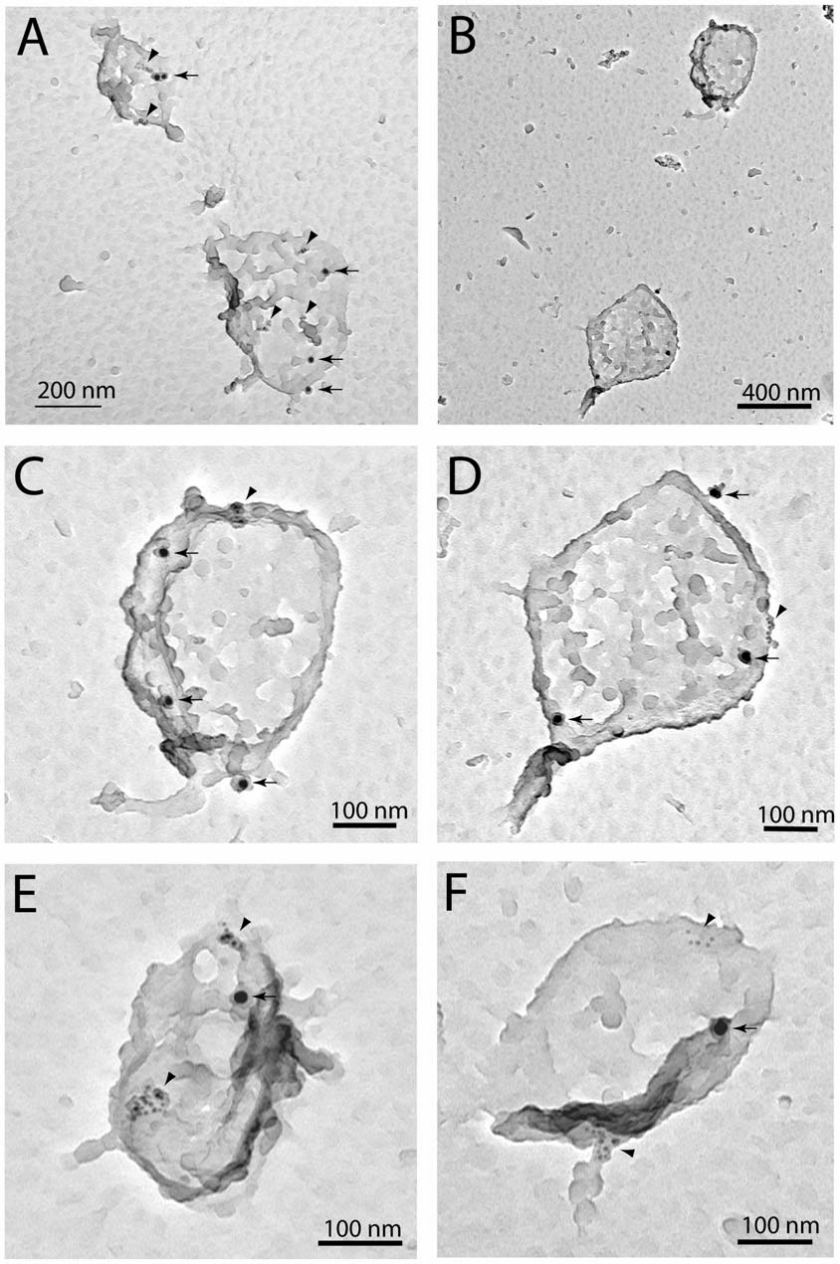
Localization of insulin receptor and caveolin-1 in endosomes by immuno-gold electron microscopy. Isolated adipocytes were incubated with insulin at 100 nM for 10 min. Cells were then homogenized and the endosomal fraction isolated. Endosome vesicles were attached to grids, immunogold-labeled against caveolin-1 (6 nm gold particles) and the insulin receptor (15 nm gold particles), lyophilized and sputtered with a 2-nm tungsten film before examination by transmission electron microscopy. C and D are blow-ups from B; arrowheads indicate patches of caveolin-1 labeling; arrows indicate insulin receptor labeling. One experiment of three with similar results is illustrated.

Transmission electron microscopy and immunogold labeling also revealed that the insulin receptor and caveolin-1 were recovered in the same vesicles in the endosome fraction (Fig. 4). It is interesting that (10 min after insulin addition) a majority of insulin receptors were detected in vesicles larger (200–400 nm) (Fig. 4) than a single caveola (ca 100 nm), indicating rapid fusion of the immediate formed endocytosed vesicles. This was further indicated by these vesicles staining for caveolin-1 in distinct patches (Fig. 4), which may reflect the organization in their caveolae of origin, where caveolin in primary adipocytes is found in the membrane proximal parts of caveolae [39].

**Figure 5:**
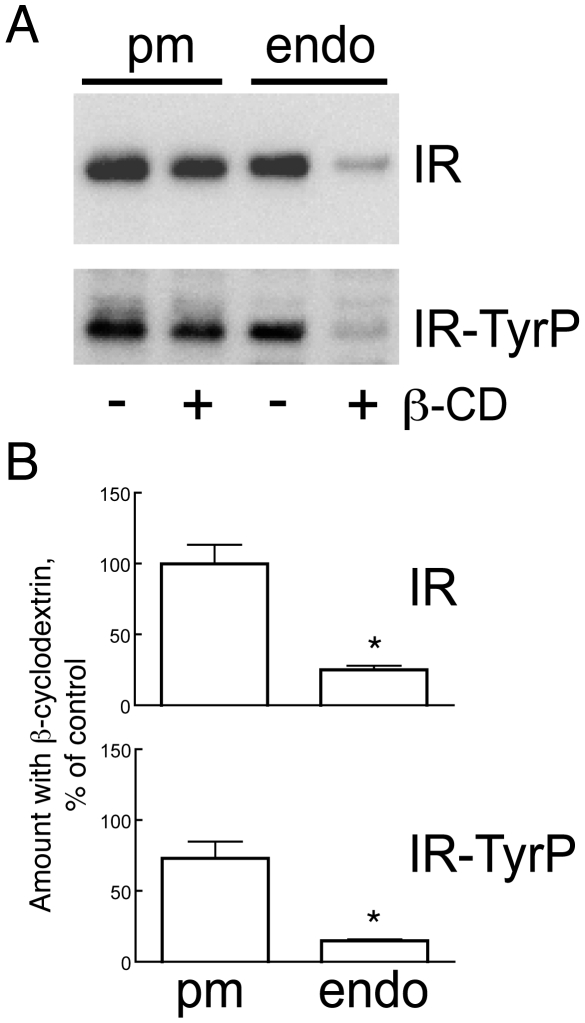
Cholesterol depletion of the cells blocks insulin receptor endocytosis. Isolated adipocytes were incubated with or without 10 mM β-cyclodextrin (β−CD) for 50 min, to deplete the plasma membrane of about 50% of its cholesterol, which completely destroys caveolae while leaving the cells intact [23]. Insulin at 100 nM was added for another 5 min. Cells were then homogenized and plasma membrane (pm) and endosomal (endo) fractions isolated. A. Equal amounts of protein were subjected to SDS-PAGE and immunoblotting with (from the top) antibodies against insulin receptor β-subunit (IR) or phosphotyrosine (indicated is insulin receptor, IR-TyrP). B. Quantitation of three experiments. Presented as amount of IR or IR-TyrP in the presence of β-cyclodextrin as % of controls without β-cyclodextrin, mean±SE. *, p<0.05 by Student’s t-test.

Caveolae are in adipocytes dependent on cholesterol for their structural and functional integrity [23], [39]. We have previously demonstrated that cholesterol depletion of rat adipocytes by β-cyclodextrin causes caveolae to flatten and disappear from the cell membrane and, in parallel, the caveolar openings on the cell surface to disappear [39]. However, insulin receptors remain in the plasma membrane and bind and respond unperturbed to insulin, but insulin signaling for phosphorylation of the downstream signal mediator IRS1 and metabolic control is blocked [22], [23]. We partially depleted the cell’s plasma membrane of cholesterol by using β-cyclodextrin and examined the insulin-induced endocytosis of the insulin receptor. In parallel we analyzed the transferrin receptor as a marker of endocytosis via clathrin-coated pits. In cells depleted of cholesterol, endocytosis of both the insulin receptor (Fig. 5) and the transferrin receptor (not shown) was blocked. Autophosphorylation of the insulin receptor in the plasma membrane remained unaffected though (Fig. 5), demonstrating the integrity of the cholesterol-depleted cells. While this is compatible with a caveolae-mediated endocytosis of the insulin receptor, cholesterol depletion does not differentiate between caveolae and clathrin coated pit-mediated endocytosis.

**Figure 6:**
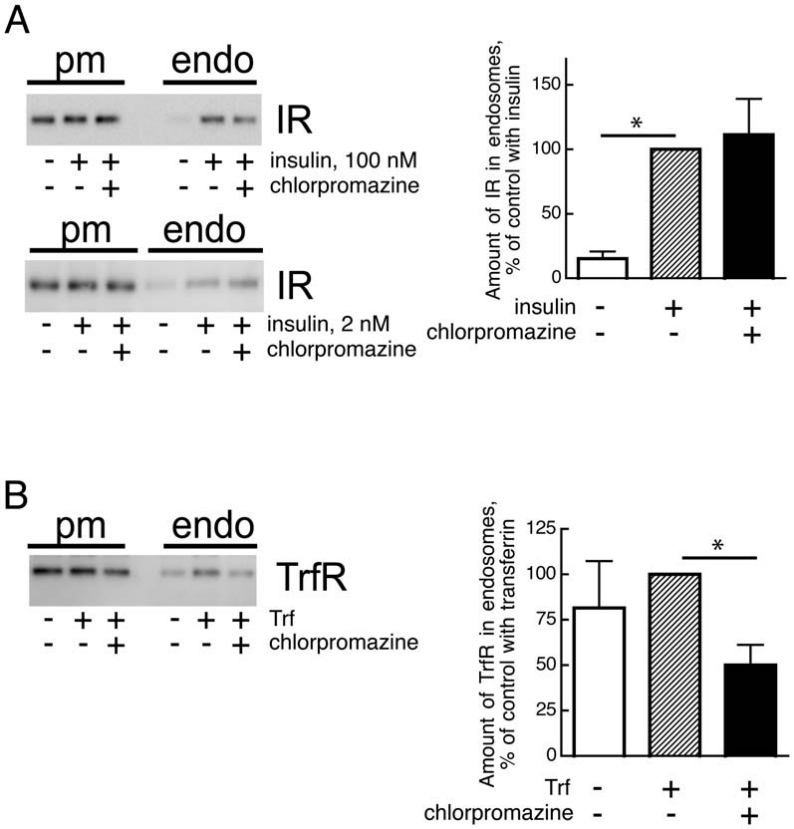
Effect of chlorpromazine on insulin receptor and transferrin receptor endocytosis. Isolated adipocytes were incubated with 50 µM chlorpromazine for 20 min when insulin at 100 nM (A), or transferrin (Trf) at 25 µg/mL (B) was added for another 10 min. Cells were then homogenized and plasma membrane (pm) and endosomal fractions (endo) isolated. Equal amounts of protein were subjected to SDS-PAGE and immunoblotting with antibodies against the insulin receptor β-subunit (IR) (A) or transferrin receptor (TrfR) (B). Results are expressed as amount of IR in the endosomal fraction as % of insulin-treated controls without chlorpromazine, mean±SE of 4 separate experiments (A); or as amount of TrfR in the endosomal fraction as % of transferrin-added controls without chlorpromazine, mean±SE of 4 separate experiments (B). *, p<0.05 by Student’s t-test.

Next, we examined the effects of chlorpromazine on insulin receptor endocytosis. Chlorpromazine is a cationic and amphiphilic compound that prevents assembly of AP-2, the clathrin adaptor protein-2, on clathrin-coated pits and thus causes a loss of clathrin-coated pit-mediated endocytosis without affecting caveolae and caveolae-mediated endocytosis [41]–[43]. Chlorpromazine treatment of the cells had no significant effect on the amount of insulin receptor recovered in the endosome fraction in response to insulin, neither at high (100 nM) nor at low (2 nM) insulin concentration (Fig. 6A), while it blocked the endocytosis of the transferrin receptor (Fig. 6B). This demonstrates that the insulin receptor was not endocytosed to a significant extent by a clathrin-coated pit-mediated process.

## Discussion

Our findings show that in response to insulin, the insulin receptor is internalized by caveolae-mediated endocytosis in primary adipocytes: (i) After stimulation with insulin the insulin receptor was co-localized with caveolin in the endosomal fraction as determined by co-immunocapture using both anti-insulin receptor and anti-caveolin antibodies, as well as by (ii) immunogold labeling and transmission electron microscopy of the endosomal fraction. (iii) Insulin stimulated the tyrosine phosphorylation and endocytosis of both the insulin receptor and caveolin in parallel. (iv) Endocytosis of the receptor was not inhibited by chlorpromazine, an inhibitor of clathrin-coated pit-mediated endocytosis.

Caveolae-mediated endocytosis of the insulin receptor is not surprising as the receptor in the plasma membrane of adipocytes has been shown to be localized in caveolae [22], [25], [44]. By electron microscopy of adipocytes, metal-labeled insulin has also been found in non-coated micropinocytotic invaginations, but not in clathrin-coated pits [45], [46], with the majority of insulin internalized via non-coated micropinocytotic invaginations [45]. The insulin receptor has, moreover, been shown to interact with caveolin in the plasma membrane [47]; and in caveolin-1 knockout mice insulin signaling is dysfunctional [48]. Failure to detect insulin receptors in caveolae [29], [30] can be explained by the solubility of the insulin receptor in non-ionic detergent [22], while many caveolar proteins, including caveolin, are detergent insoluble. The group of Carpentier has earlier reported insulin binding in clathrin-coated pits in various cell types, e.g. [28], but has also recently found that the insulin receptors are located in caveolae of 3T3-L1 adipocytes [25]. Without considering endocytosis of the receptor, it has also been reported that the insulin receptor is not co-localized with caveolin in intracellular membranes from adipocytes, except following treatment with insulin [30].

The principal alternative for endocytosis of the insulin receptor is via clathrin-coated pits [1], [19], [49]. Our findings, that chlorpromazine blocked endocytosis of the transferrin receptor without effect on internalization of the insulin receptor, argue against that. Clathrin-coated pits, moreover, are present at low levels in primary adipocytes (membrane area occupied by clathrin-coated pits is <2% of caveolae membrane area) [39], [45], [46]. Hence it is not likely that the insulin receptors are endocytosed to any large extent through this route in adipocytes, although endocytosis through clathrin-coated pits can be a major route in other cell types, such as hepatocytes with little caveolae (see below). The small delay between insulin receptor autophosphorylation/activation and receptor appearance in the endosome fraction of adipocytes would preclude a relocation of insulin receptors from caveolae to clathrin-coated pits for endocytosis, as has been suggested for the epidermal growth factor receptor [50]. At high concentrations of EGF a significant fraction of the EGF receptor has been found to relocate from clathrin coated pits to a non-clathrin, lipid raft-dependent route for internalization [51]. However, internalization of the insulin receptor was not inhibited by chlorpromazine, neither at low nor at high insulin concentrations, which indicates that in adipocytes the insulin receptors are not internalized by different routes at different concentrations of insulin.

Endocytosis of insulin receptors in adipocytes reached a maximum with t_1/2_<3 min, comparable to the time-course reported for intracellular accumulation of radiolabeled insulin [52] or metal-labeled insulin [46] in rat adipocytes. This can be compared with the situation in Fao hepatoma cells, where insulin receptors are internalized with t_1/2_ = 10.5 min [13], presumably via clathrin-coated pits. Our findings indicate that there was very little basal or default endocytosis of the insulin receptor in primary adipocytes and that receptor autophosphorylation was required for endocytosis, which is compatible with the localization of the receptor in caveolae that exhibit a low constitutive endocytosis rate [31], [35]. Insulin transport over the blood vessel endothelium appears to be via a transcellular route in some tissues and this may involve caveolae transcytosis. It was thus demonstrated that the cholesterol-sequestering agent filipin inhibited insulin transport across monolayers of cultured bovine aortic endothelial cells [53]. Recently, Wang et al. [54] demonstrated transcytosis of insulin in vivo in skeletal muscle and in cultured bovine aortic endothelial cells, which appeared to involve the insulin receptor and caveolae. FITC-insulin was endocytosed by the endothelial cells together with the insulin receptor and caveolin, suggesting a caveolae-mediated endocytosis/transcytosis of the insulin-occupied insulin receptor in transendothelial transport for signaling in the interstitial muscle fibers.

Cholesterol depletion of the plasma membrane, which inhibits downstream signaling to metabolic control without affecting insulin binding or autophosphorylation of the receptor [23], [55], blocked endocytosis of the insulin receptor, which can be interpreted as a dependence on endocytosis for efficient downstream signaling by the insulin receptor. Clearly, more research is required to clarify the function of receptor endocytosis in insulin signaling.

It is interesting that the insulin receptor was endocytosed as fast as it was phosphorylated in response to insulin, such that the ratio of phosphorylated to total receptor increased much more rapidly in the endosomal fraction compared with the plasma membrane, which is in line with an earlier report [10]. This indicates that those caveolae containing insulin-activated receptors were preferentially or exclusively endocytosed, which is compatible with the dependence of insulin receptor internalization on insulin receptor autophosphorylation and independence of downstream activation of IRS and phosphatidylinositol-3 kinase [8], [17], [18]. In case of insulin causing a more general stimulation of caveolar endocytosis, phosphorylated and non-phosphorylated forms of the insulin receptor would have been endocytosed at a more or less similar rate. Regulated endocytosis of a specific subset of caveolae is in line with our earlier identification of specific subclasses of caveolae with distinct lipid and protein contents as well as biological functions [56]–[58]. The endocytosed receptor was maximally phosphorylated after about 2 min and then increasingly dephosphorylated, which is compatible with the intracellular, endoplasmic reticulum, localization of the major insulin receptor phosphotyrosine protein phosphatase PTP1B [59]–[61].

A dependence on phosphorylation of caveolin-1 at tyrosine(14) for caveolae-mediated endocytosis has been reported [38], reviewed in [62], and has been linked to induction of the endocytosis process [36]. Phosphorylated caveolin has been found to specifically interact with the protein Grb7 [63], but the further effects of this particular interaction is not known.

In response to insulin caveolin was phosphorylated in conjunction with endocytosis of the insulin receptor. It has earlier been demonstrated that the insulin receptor phosphorylates caveolin on tyrosine(14) in 3T3-L1 adipocytes [63]–[66]. The failure of genistein to inhibit the insulin-stimulated phosphorylation of caveolin-1 indicates a direct phosphorylation of caveolin-1 by the insulin receptor, as the insulin receptor was not affected by genistein, while src-kinase, which is known to phosphorylate caveolin-1, is sensitive to inhibition by genistein [63]–[65], [67]. The low extent of phosphorylation of caveolin-1 in response to insulin is a predictable consequence of a direct phosphorylation of caveolin-1 by the insulin receptor, as only a small fraction of all receptors was activated. Endocytosis of the insulin receptor has been demonstrated after several hours of treatment with the tyrosine phosphatase inhibitor vanadate in IM9 lymphocytes [68]. However, we found that, in spite of a higher degree of phosphorylation of caveolin-1 in the presence of the protein tyrosine phosphatase inhibitor vanadate than in response to insulin, phosphorylation of the insulin receptor and receptor endocytosis was not affected. This could suggest that if caveolin-1 phosphorylation is involved in triggering caveolae and insulin receptor endocytosis, it is not sufficient for endocytosis of the receptor. It should be borne in mind though that vanadate may not have affected phosphorylation of enough caveolin-1 in those caveolae that harbor the insulin receptor. Alternatively, it is possible that caveolin-1 in the insulin receptor-containing caveolae are strictly under control by insulin receptor-catalyzed phosphorylation. It will be an important challenge to elucidate the precise mechanisms and role of caveolin tyrosine phosphorylation in the regulated endocytosis of the insulin receptor by caveolae.

Phosphorylation of caveolin at tyrosine(14) in NRK cells, correlates with the intracellular appearance of vesicles with patches of caveolin labeling, as seen by immunogold electron microscopy [69]. This is similar to our finding of caveolin- and insulin receptor-containing vesicles, with patches of caveolin-1-labeling, in the endosome fraction. Finding the endocytosed receptor in such caveolin-containing vesicles suggests that these may represent a caveolin-containing endosomal compartment that in adipocytes may correspond to caveosomes, as found for SV40 virus uptake by caveolae en route to the endoplasmic reticulum in CV-1 cells [70]. Caveolin has in MDCK cells also been found in recycling endosomes that exhibit an extended tubular morphology in electron micrographs [71].

In conclusion, our findings show that in the early phase of insulin signaling in primary adipocytes, the insulin receptor is primarily endocytosed in a caveolae-mediated process involving tyrosine-phosphorylation of caveolin-1. It remains, however, to further characterize the insulin receptor-containing endosomes, their composition, their subsequent fate, as well as their relation to caveosomes.

## Materials and Methods

### Materials

Caveolin-1 monoclonal antibodies (610058, or 610493 for immunogold-labeling), anti-PY14-caveolin-1 monoclonal antibodies, and anti-phosphotyrosine (PY20) monoclonal antibodies were from Transduction Laboratories (Lexington, KY, USA). Anti-insulin receptor β-chain rabbit polyclonal antibodies were from Santa Cruz Biotechnology (Santa Cruz, CA, USA). Monoclonal anti-transferrin receptor antibodies were from Zymed Laboratories Inc. (South San Francisco, CA, USA). Harlan Sprague Dawley rats (140–160 g) were obtained from B&K Universal, Sollentuna, Sweden. The animals were treated in accordance with Swedish animal care regulations.

### Isolation and incubation of adipocytes

Adipocytes were isolated by collagenase (type 1, Worthington) digestion [72]. Cells were kept in Krebs-Ringer solution (0.12 M NaCl, 4.7 mM KCl, 2.5 mM CaCl_2_, 1.2 mM MgSO_4_, 1.2 mM KH_2_PO_4_) containing 20 mM Hepes, pH 7.40, 1% (w/v) fatty acid-free bovine serum albumin, 100 nM phenylisopropyladenosine, 0.5 U.ml^−1^ adenosine deaminase with 2 mM glucose, at 37°C, except as indicated, on a shaking water bath.

### Isolation of plasma membrane and endosome fractions

Adipocytes were homogenized in 10 mM Tris-HCl, pH 7.4, 1 mM EDTA, 0.5 mM EGTA, 0.25 M sucrose, 25 mM NaF, 1 mM Na_2_-pyrophosphate, with protease inhibitors; 10 µM leupeptin, 1 µM pepstatin, 1 µM aprotinin, 4 mM iodoacetate, and 50 µM phenylmethylsulfonyl fluoride, using 2 strokes with a motor-driven loose-fitting Potter-Elvehjem teflon/glass homogenizer at room temperature. Subsequent procedures were carried out at 0–4°C. Cell debris and nuclei were removed by centrifugation at 1000×g for 10 min. A plasma membrane-containing pellet, obtained by centrifugation at 16,000×g for 20 min, was resuspended in 10 mM Tris-HCl, pH 7.4, 1 mM EDTA and protease inhibitors. Purified plasma membranes were obtained by sucrose density gradient centrifugation on a 1.12 M sucrose cushion at 140,000×g for 60 min (referred to as plasma membrane fraction), as detailed and characterized in [73], [74].

The fraction containing the internalized receptor, referred to as the endosome fraction, was obtained by sucrose density gradient centrifugation of the 16,000×g supernatant on a 0.67 M and 1.05 M discontinuous sucrose gradient at 100,000×g for 75 min. The material collected from the interphase between the 0.67 and 1.05 M sucrose is referred to as the endosome fraction [12]. To minimize a risk that vesicles aggregate, fuse, or exchange components, cells were very mildly homogenized by two strokes of a loose-fitting Potter-Elvehjem homogenizer and membranes were never pelleted during the entire procedure. We also avoided all means of concentrating the endosomal vesicles, by centrifugal pelleting or otherwise, leaving the vesicles in a dilute solution to preserve their integrity.

### Immunocapture

The endosome fraction (ca 25 µg) was incubated under rotation with antibodies against the insulin receptor (rabbit polyclonal) or tyrosine(14)-phosphorylated caveolin (mouse monoclonal) for 1 h at room temperature in the presence of protease inhibitors. Magnetic Dynabeads M-450 (Dynal, Oslo, Norway) with coupled anti-rabbit or anti-mouse antibodies, respectively, were then added and incubated for 2 h under rotation at room temperature. After extensive washing, immunocaptured endosomal vesicles were treated for SDS-PAGE.

### Electron microscopy

Endosome membranes were collected on carbon formvar covered nickel grids by placing a drop of the endosome fraction on the grids. After equilibration in 0.1 M cacodylate buffer with 0.1 M sucrose for 15 min on ice, grids were prefixed in 3% paraformaldehyde +0.05% glutaraldehyde in the same buffer for 15 min, washed, and blocked in 137 mM NaCl, 2.7 mM KCl, 11.8 mM phosphate, pH 7.5 (PBS) with 0.15 M glycine and then in PBS with 3% (w/v) bovine serum albumin (BSA-c, Aurion, The Netherlands), 1% (v/v) normal goat serum, and 0.1% (w/v) gelatin for 40 min at 37°C. The grids were incubated with mouse anti-caveolin-1 monoclonal antibody and rabbit anti-β-subunit insulin receptor polyclonal antibodies overnight at 4°C. Primary antibodies were detected with 6 nm and 15 nm, respectively, colloidal gold-conjugated secondary antibodies (Aurion) after incubation overnight at 4°C. Controls were without primary antibody. The membranes were rinsed and fixed in 2% glutaraldehyde for 10 min followed by 1% OsO_4_ for 15 min in 0.1 M cacodylate buffer, with 0.1 M sucrose, pH 7.5, at room temperature. Grids were rinsed with water, frozen, lyophilized, and coated with 2 nm tungsten by magnetron sputtering directly in the freeze-dryer [75]. Transmission electron microscopy was done with Jeol EX1230 TEM (Tokyo, Japan).

### Additional procedures

Protein was determined with the Micro BCA protein assay kit from Pierce. Bovine serum albumin was used as reference. For immunoblotting, protein was separated by SDS-PAGE, transferred to a polyvinylidene difluoride blotting membrane (Immobilone-P, Millipore, Bedford, MA, USA) and incubated with indicated primary antibodies. Bound antibodies were detected using Renaissence+ (PerkinElmer Inc., Shelton, CT, USA) or ECL (Amersham Biosciences, Amersham, UK) with horseradish peroxidase-conjugated anti-IgG as secondary antibody. Blots were evaluated by chemiluminescence imaging (Las 1000, Fuji, Japan).
